# Testing Seven Hypotheses to Determine What Explains the Current Planthopper (Fulgoridae) Geographical and Species Richness Patterns in China

**DOI:** 10.3390/insects11120892

**Published:** 2020-12-17

**Authors:** Zheng-Xue Zhao, Lin Yang, Jian-Kun Long, Zhi-Min Chang, Zheng-Xiang Zhou, Yan Zhi, Liang-Jing Yang, Hong-Xing Li, Yong-Jin Sui, Nian Gong, Xiao-Ya Wang, Xiang-Sheng Chen

**Affiliations:** 1 Institute of Entomology, Guizhou University, Guiyang 550025, China; zzx611324@163.com (Z.-X.Z.); yanglin6626@163.com (L.Y.); Zhouzx2016@163.com (Z.-X.Z.); zhiyan0428@163.com (Y.Z.); ljyang2018@163.com (L.-J.Y.); lhx5340@163.com (H.-X.L.); syjkk2016@163.com (Y.-J.S.); 18285180546@163.com (N.G.); wangxy541@163.com (X.-Y.W.); 2 Provincial Special Key Laboratory for Development and Utilization of Insect Resources of Guizhou, Guizhou University, Guiyang 550025, China; 3 Guizhou Key Laboratory for Agricultural Pest Management of Mountainous Region, Guizhou University, Guiyang 550025, China; 4 College of Animal Science, Guizhou University, Guiyang 550025, China; Longjiankun123@163.com (J.-K.L.); cczzmm111@126.com (Z.-M.C.)

**Keywords:** ambient energy, biogeography, dispersal ability, historical climate stability, Last Glacial Maximum, planthoppers, productivity, species richness

## Abstract

**Simple Summary:**

Although there are many described species and ample geographical distribution data available on planthoppers (Fulgoridae) in China, the research on the underlying mechanisms of macro-scale richness patterns is still scant. To contribute to unraveling these mechanisms, we tested seven hypotheses related to contemporary environments and historical climate stability by relating the species richness to 15 environmental variables. The historical climate stability, ambient energy, and productivity hypotheses are superior to other hypotheses in explaining the current richness patterns of planthoppers. Based on these main hypotheses, we narrowed the mechanisms underlying contemporary planthopper distribution in China. Furthermore, other factors not included in this study (i.e., orogenic processes and geological isolation) may significantly contribute to richness patterns identified here.

**Abstract:**

Although many hypotheses have been proposed to understand the mechanisms underlying large-scale richness patterns, the environmental determinants are still poorly understood, particularly in insects. Here, we tested the relative contributions of seven hypotheses previously proposed to explain planthopper richness patterns in China. The richness patterns were visualized at a 1° × 1° grid size, using 14,722 distribution records for 1335 planthoppers. We used ordinary least squares and spatial error simultaneous autoregressive models to examine the relationships between richness and single environmental variables and employed model averaging to assess the environmental variable relative roles. Species richness was unevenly distributed, with high species numbers occurring in the central and southern mountainous areas. The mean annual temperature change since the Last Glacial Maximum was the most important factor for richness patterns, followed by mean annual temperature and net primary productivity. Therefore, historical climate stability, ambient energy, and productivity hypotheses were supported strongly, but orogenic processes and geological isolation may also play a vital role.

## 1. Introduction

Species richness, the simplest biodiversity index, is unevenly distributed on the Earth’s surface [1,2,3]. Identifying and understanding the factors and mechanisms responsible for the underlying spatial species richness variation have been major goals in biogeography and ecology since the 18th century [4,5,6,7]. This knowledge can provide valuable insights regarding biodiversity conservation in the context of global climate change [8,9,10]. To understand the mechanisms underlying species richness geographical patterns, numerous hypotheses have been proposed in the past few decades that are divided into modern environmental conditions (climate, habitat heterogeneity, and human activities) and evolutionary/historical processes (speciation, extinction, and dispersal).

Initial hypotheses were established based on the statistical relationships between modern environments and species richness patterns [1,11,12,13,14]. Among these, the energy hypothesis, including ambient energy, water–energy dynamics, and productivity versions received the most attention when originally proposed and are still the leading hypotheses [15,16,17,18]. Unfortunately, it is difficult to obtain robust mechanistic frameworks based only on modern explanations [19]. This is because species have evolved in the past in specific locations, and thus historical factors will inevitably affect the species richness distribution patterns [20]. In other words, the statistical relationships between modern environments and species richness relate poorly with the historical components underlying richness patterns. Indeed, early biogeographers observed the influence of historical climate factor on species richness, and the historical climate stability hypothesis was established on this basis. However, correlative studies claim that this hypothesis is of lower importance than the modern climate one [20,21,22]. It is noteworthy that the overwhelming role of the contemporary environment hypotheses in previous studies may not be correct, largely because the authors used coarse estimates of historical climates (e.g., time since the retreat of ice), resulting in a statistical bias in favor of modern explanations [8]. In recent years, with rapid climate model development for producing high-resolution palaeoclimatic data, a growing number of studies have shown that historical climate changes, particularly climate changes since the Last Glacial Maximum (LGM), are important in explaining current richness patterns [23,24,25]—sometimes exceeding modern environments [10,26,27]. Nevertheless, despite many efforts, there is still no consensus on the relative importance of various hypotheses representing contemporary environments and historical climate stability in shaping the diversity patterns, especially insects.

The planthopper superfamily Fulgoroidea (Insecta: Hemiptera) is one of the most numerous herbivorous insect species. The bizarre morphology is represented in many members of planthoppers, and a few species have been found with rows of cuticular gear (cog) teeth on the hind legs [28,29]. These characteristics has drawn great attention from biologists. Most planthoppers feed on the phloem tissue of woody or herbaceous plants, while a few feed on fungi, horseradish, mosses, or ferns [30,31]. Planthoppers are major agricultural pests, especially *Sogatella furcifera*, *Nilaparvata lugens*, and *Laodelphax striatellus*. They feed on a variety of crops, such as corn, wheat, barley, and rice [32]. Furthermore, some species can become vectors for plant pathogens, including bacteria, viruses, and phytoplasmas [33,34,35,36]. Planthoppers are distributed worldwide, and they occupy major biomes, such as tropical rainforests, deserts, and grasslands [32], and represent an excellent model for biogeography studies [37]. However, the statistical explanation for the geographic species richness distribution is poorly understood, especially in China.

China occupies a huge territory—broad latitudinal range, complex terrain, and a diverse climate from south to north (significant warm to cold gradient) and east to west (significant wet to dry gradient) [38,39]. The evolutionary history in China is significantly different from that in Europe and North America [40]. One obvious difference is that China was less affected by historical glaciation. This provides a good opportunity to understand what drove the factors and mechanisms of richness patterns in different biogeographic regions. Furthermore, biodiversity surveys have been conducted across the country in recent decades, providing basic research data for biogeographical comparisons. Cumulatively, China is an ideal region to investigate the richness–environment relationships. This study aimed to investigate the relationships between planthopper richness and environmental variables accounting for species richness geographical patterns testing seven hypotheses across China (Table 1) in an attempt to identify the most robust hypotheses explaining planthopper richness.

## 2. Materials and Methods

### 2.1. Species Distributional Data

We compiled a database of 14,722 distribution records for 1335 planthopper species, based on mainstream literature, books, MD/PhD theses, zoological records, China Knowledge Resource Integrated Database, GBIF website (https://www.gbif.org/), and specimens examined from the following museums: Zoological Museum, Institute of Zoology, Chinese Academy of Sciences; Institute of Entomology, Guizhou University; the insect collection of China Agricultural University, Hebei University, Nankai University, and Dali University; Entomological Museum, Northwestern A & F University; Shanghai Institute of Entomology, Chinese Academy of Sciences; Tianjin Museum of Natural History; and Taiwan Agricultural Research Institute. We obtained the locations at the city, county, or township levels from the original source. Locations included precise geographic coordinates at the original source, and the coordinates data was directly used in subsequent analysis; however, locations that had no geographic coordinates were georeferenced by identifying the corresponding administrative center in Google Earth. A 1° × 1° grid size (~100 km × 100 km) was employed to visualize the species richness patterns, considering the resolution of the distribution data and appropriateness for richness–environment relationship assessment.

### 2.2. Environmental Data

We related the planthopper species richness to the 15 environmental variables listed in Table 1 (what follows are expanded definitions): (i) ambient energy, mean annual temperature (MAT), and annual potential evapotranspiration (PET); (ii) water–energy dynamics, mean annual precipitation (MAP), and annual actual evapotranspiration (AET); (iii) environmental stability, temperature annual range (TAR), and precipitation seasonality (PS); (iv) productivity, normalized difference vegetation index (NDVI), and net primary productivity (NPP); (v) habitat heterogeneity, elevation range (ER; the difference between the maximum and minimum elevation in 1° grid), slope (SP), and topographical roughness (TR; see Equation (1)); (vi) human activities, human population (HPP), and gross domestic product (GDP); and (vii) historical climate stability, MAT and MAP change since the LGM (defined as MAT anomaly = |modern_MAT_ − LGM_MAT_| and MAP anomaly = |modern_MAP_ − LGM_MAP_|). The past climate model uncertainty was considered; MAT and MAP in LGM were represented by computing the mean of two available LGM simulations from the Model for Interdisciplinary Research on Climate Earth system (MIROC-ESM) and Community Climate System Model version 4 (CCSM4) models.

(1) $$\mathrm{TR}=1/\mathrm{Cos}\left(\left[\mathrm{Slope}\right]\;*\;3.14159/180\right)$$

where “Slope” is the slope of each 1° grid.

We obtained modern and LGM MAT/MAP, TAR, and TS from the WorldClim database (http://www.worldclim.org) using the data with a spatial resolution of 30 arc-seconds (~1 km^2^). PET and AET at 30 arc-seconds (~1 km^2^) spatial resolution were obtained from the CGIAR-CSI database (http://www.cgiar-csi.org). NDVI, NPP, HPP, and GDP were downloaded from the Resource and Environment Data Cloud Platform (http://www.resdc.cn/) with a spatial resolution of 1 km^2^. Elevation data were downloaded from CGIAR SRTM (http://srtm.csi.cgiar.org/) at 30 arc-seconds (~1 km^2^) spatial resolution, which was used to calculate ER, SP, and TR. To obtain the variable values in a 1° grid, we calculated the average of all 1 × 1 km^2^ grid cells using ArcGIS 10.5 (ESRI, Redlands, CA, USA).

### 2.3. Statistical Analyses

As sampling bias may distort the geographic richness patterns [41] and further affect the reliable richness–environment relationship evaluation [42], assessing the quality of the collected distribution data is the first step in macroecological pattern analyses and inferences. Here, we employed a bootstrap species accumulation curve to assess data completeness across the study region. The presence (1) or absence (0) matrix for each species in each 1° grid was constructed and was analyzed using EstimateS 9.1 with 100 randomizations [43]. Furthermore, a linear regression was developed using the square-root transformed number of records as predictor variable and square-root transformed numbers of richness as response variable in each 1° grid cell, which aims to evaluate the species richness completeness in each grid. The *p*-value was reported using geographically effective degrees of freedom [44], evaluated using Spatial Analysis in Macroecology 4.0 [45].

We used ordinary least squares (OLS) models to examine the relationships between richness and each environmental variable. Also, spatial error simultaneous autoregressive (SAR_err_) models with spatial weights matrix row-standardization were fitted to account for the spatial autocorrelation in model residuals. The SAR_err_ models were selected based on the lowest value of Akaike information criterion corrected for small sample sizes (AICc). Pseudo *R*^2^ in the SAR_err_ models was defined as the squared Pearson correlation between predicted and observed (i.e., true) values [46]. The model averaging approach, a procedure to account for the model selection uncertainty, was used to evaluate the relative roles of environmental variables recognized as measures of the seven richness pattern hypotheses. We excluded PET, TR, and MAP anomaly in the model averaging because they did not significantly predict species richness in a univariate regression models (*p* > 0.05; see SAR_err_ analysis in Table 2). To assess the significance of the 12 predictor variables’ multicollinearities, we computed variance inflation factors (VIFs). The VIFs for all remaining variables were <20 and were entered in the model averaging [18]. All possible variable combinations were built models, and these models were ranked based on the ascending AICc value. The importance of each variable was represented by the AIC weighted sums for the models in which they appear.

Before regression analyses, TAR, PS, ER, TR, GDP, HPP, and MAT anomaly were log10-transformed, and AET, SP, and MAP anomaly were square root-transformed. The remaining variables are not transformed. We additionally standardized (mean = 0 and standard deviation = 1) all variables. Statistical analyses were run in R 3.6.1 [47] using the MuMIn [48] and spdep [49] packages.

## 3. Results

### 3.1. Planthopper Taxonomic Diversity

**Figure 1:**
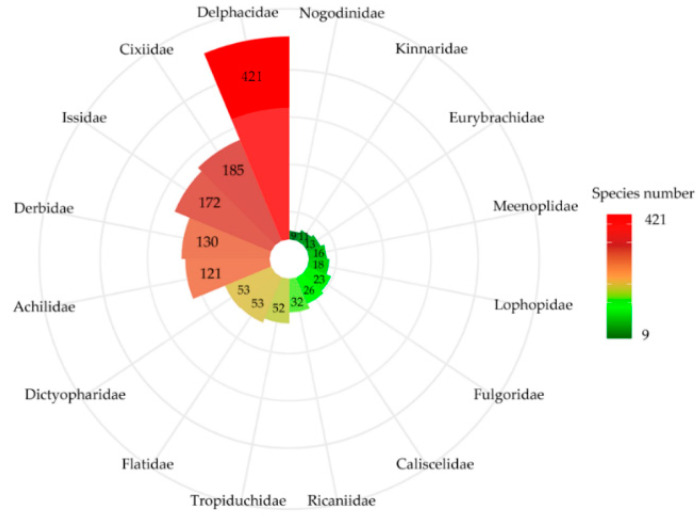
Species number in each planthopper family.

We worked with 1335 planthopper species (approximately 9.7% of the global species) with available distribution data belonging to 16 families. There were several different species number represented in each planthopper family, and several species-rich families were found (Figure 1). Delphacidae has the largest species number (421 species) accounting for 31.5% of the total number of species. This was followed by Cixiidae (185 species, 13.8% of all species), Issidae (172 species, 12.8% of all species), Derbidae (130 species, 9.7% of all species), and Achilidae (121 species, 9% of all species). The species number in the other families was ≤ 53.

### 3.2. Species Richness–Environment Relationships

**Figure 2:**
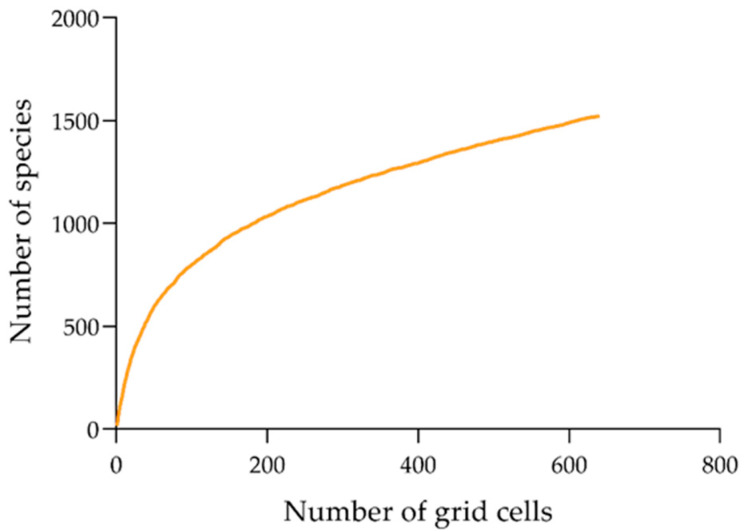
Species bootstrapped accumulation curve.

**Figure 3:**
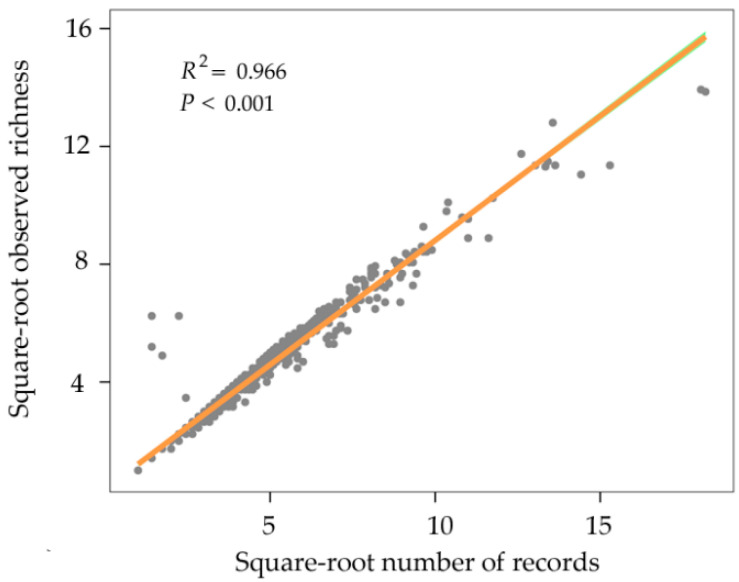
Linear regression (y = 0.844x + 0.367) for the square-root number of records and square-root number of richness in a 1° grid.

**Figure 4:**
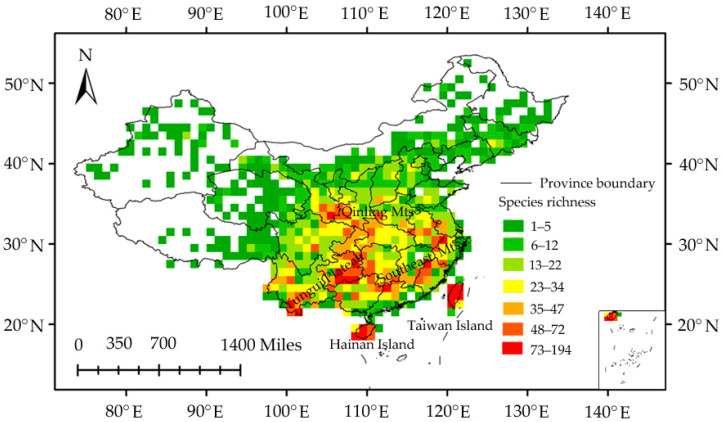
Planthopper species richness patterns in a 1° grid size.

The “true” species number using species bootstrap accumulation curves was 1526 (Figure 2), and the data completeness degree across the region was 87.5%. Moreover, the observed species richness was >82.7% of the predicted species richness based on the linear regression (Figure 3). These results reflect a good sampling degree, allowing one to draw reliable conclusions between the richness patterns and relative environmental factor roles that regulate these patterns. Overall, spatial species richness patterns were unevenly distributed, with few species in northern China and a high number of species in central and southern China. Specifically, the Yungui Plateau, the Qinling Mountains, the Southeast Mountains, the Taiwan Island, and the Hainan Island showed the highest number of species (Figure 4).

**Figure 5:**
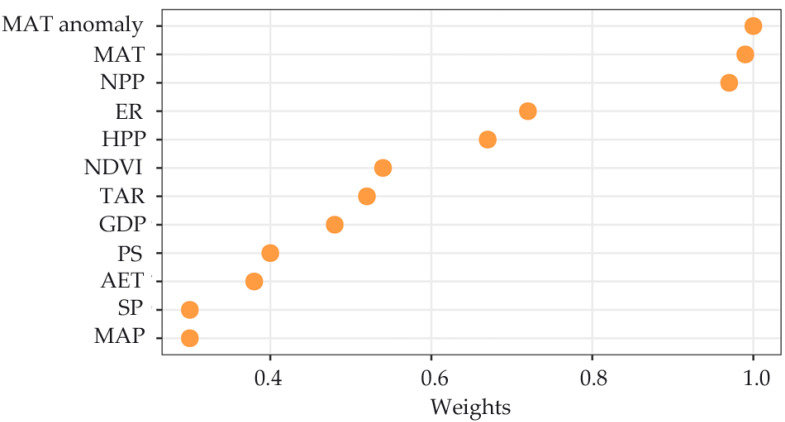
The Akaike information criterion (AIC) weighted sums for each environmental variable.

The OLS models showed that species richness negatively correlated with TAR, PS, and MAT anomaly. However, species richness positively correlated with other variables (i.e., MAT, MAP, AET, NDVI, NPP, ER, SP, HPP, and GDP). PET, TR, and MAP anomaly have no significant correlation with species richness (Table 2). SAR_err_ models revealed similar patterns that were identified in the OLS models (Table 2). Model averaging analyses showed that the MAT anomaly (historical climate stability) had the largest influence on species richness, followed by MAT (ambient energy) and NPP (productivity) (Figure 5). ER (habitat heterogeneity) and HPP (human activities) showed moderate contributions to species richness. All variables that are associated with water–energy dynamics and environmental stability had little effect on richness patterns (Figure 5).

## 4. Discussion

Paleoclimate changes caused by the Earth’s orbit have significantly altered the geographical distribution of species [50,51,52]. Consequently, the colder climate forced several species to survive in restricted glacial refuges. However, once the environmental conditions improved, many but not all species migrated from the refuges to the formerly glaciated lands [53,54]. In some respects, the modern species distribution is still controlled by post-glacial recolonization [55]. The historical climate change effect on contemporary species richness patterns depends on the ability to recolonize after the glacial period. Highly mobile species can track climate changes, whereas species with low dispersal ability have a harder time adapting [8,9,56] and leave a stronger historical signal in their contemporary distribution patterns.

**Figure 6:**
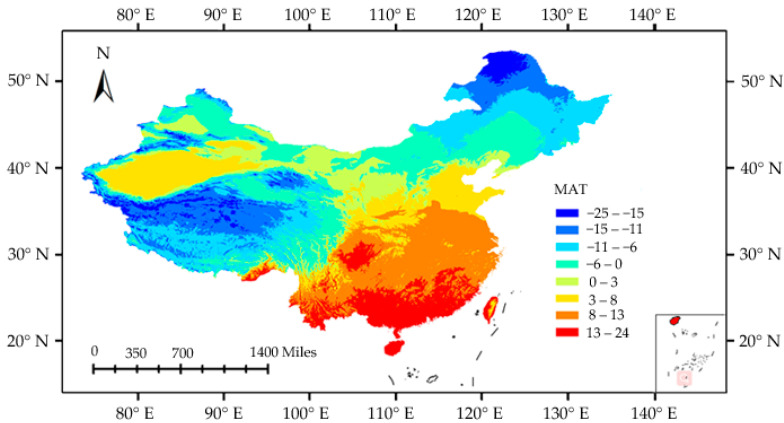
The LGM mean annual temperature (MAT) in China.

The MAT anomaly has the strongest influence on richness patterns, with a negative correlation. Our results are consistent with the historical climate stability hypothesis, which claims that areas with long-term stable climates are conducive to persistence, speciation, and low extinction rates [50,52]; all these variables promote high species richness. Planthoppers are insects with reduced dispersal ability [37], which correlates with their inherently poor ability to track climate change. Previous studies have shown the low-temperature effect on planthopper mortality and survival. For example, the *Nilaparvata lugens* nymph mortality can reach 60% at 17.5 °C, 63.7% at 17 °C, and 100% at 13 °C [57,58]. The *Sogatella furcifera* nymph survival rate is only 10% at 17.5 °C [59]. These findings provide some evidence for planthopper cold tolerance and suggest that a large portion of planthopper species have gone extinct during the LGM, with only a few species surviving in northern China where most areas were below 0 °C (Figure 6). On the contrary, planthoppers living in the south were relatively less affected by the low LGM temperature and continued to survive. Furthermore, the long-term stable climate increased specialization, speciation rates, and novel clade evolution [50,52]. After the glacial period, the planthoppers in the south gradually migrated but did not completely occupy northern China owing to their low dispersal ability. These results show that historical climate changes can take precedence over modern environments in regard to planthopper distribution in China. Similarly, previous studies also observed that historical climate changes dominate low dispersal plant richness patterns in Europe and China [10,56]. Together, our results and other studies (e.g., [10,27,56]) suggest that dispersal ability is a critical factor for assessing the relative roles of contemporary versus historical factors in species distribution.

The idea of species richness spatial patterns determined by the available energy amount can be traced back to the early days of biogeography [60]. Higher energy availability promotes and maintains higher species richness in a given area [11,14,19,61]. The most common explanation for positive energy–richness is that a larger number of individuals in a population reduce the risk of stochastic extinction [62,63,64]. MAT and NPP have a strong and positive relationship with species richness (Table 2), supporting the ambient energy and productivity version of the energy hypotheses. The ambient energy hypothesis considers that species richness is directly regulated through an ambient or solar energy effect (often quantified in temperature) on the organism’s physiology [5,65,66,67]. For example, ectotherm (e.g., insects) reproduction is more efficient in environments with higher temperatures [67], and an increase in temperatures can promote certain behaviors, such as the number of ovipositions in *Sogatella furcifera* and *Nilaparvata lugens* [68,69,70]. Although the productivity hypothesis also claims that energy availability influences species richness, it focuses on the energy flowing in the food web (chemical energy) rather than the total energy going into a given region, which is more of an indirect effect on species richness. The high correlation between planthoppers and plant productivity is logical as these insects are herbivores. In our study, NDVI was a weaker predictor than NPP, which is consistent with results reported by Qian et al. [71] looking at amphibian and reptile species richness in China. This difference may be because NPP is a better representation of primary production. The water–energy hypothesis is less important than the ambient energy hypothesis. This is consistent with the relative role of the two hypotheses in regulating spatial invertebrate species richness patterns across latitudes [14], considering that China includes large regions of temperate and boreal climates [72].

Habitat heterogeneity has a significant impact on the geographical species richness patterns in different study areas and taxonomic taxa, sometimes becoming the most important driving factor [13,73,74]. Habitat heterogeneity promotes species richness by influencing ecological, historical, and evolutionary variables [75]. Although our results show that the habitat heterogeneity represented by ER and SP was positively associated with richness patterns, it was not an important factor as found in other studies related to insect richness–environment relationships across China [27,76,77,78]. Our results, together with previous findings, may imply that habitat heterogeneity does not dominate the insect richness patterns across China. The positive relationship between human activities and species richness is mediated by productivity [79,80,81]. The relatively low human activity role in this study may be related to the wide geographical study area, based on a previous study [82]. TAR and PS, two measures of short-term climate stability, also contributed weakly to regulating spatial species richness variation, indicating little support for the environmental stability hypothesis.

High richness of planthoppers was identified in mountainous areas (Figure 4). Nevertheless, the high species diversity in these areas cannot be simply explained by the ecological and historical processes found there. We suggest that orogenic processes and geological isolation also play an important role. In China, many studies have found that orogenic processes are an important driving force in promoting rapid insect speciation, such as aphids [83], moths [84], and Blattodea insects [85]. Additionally, the high species richness in the Taiwan and Hainan Islands (typical mountainous islands) is also attributed to the high speciation and diversification caused by their mainland isolation, as suggested in previous studies [54,86,87,88,89]. Therefore, it will be necessary to use a molecular phylogeny to test the importance of these factors in the modern patterns of planthopper richness in future works.

## 5. Conclusions

We investigated richness patterns of planthoppers in China and assessed the relative importance of seven proposed hypotheses related to ambient energy, water–energy dynamics, productivity, environmental stability, habitat heterogeneity, human activities, and historical climate stability in these patterns. High species richness was identified in the Yungui Plateau, the Qinling Mountains, the Southeast Mountains, the Taiwan Island, and the Hainan Island. The MAT change since the Last Glacial Maximum was the most influential variable in shaping planthopper richness patterns, which may be related to their poor post-glacial recolonization and low dispersal ability. We also observed that the MAT and NPP are two significant variables. By contrast, other environmental variables showed relatively weak influence. Our results support the historical climate stability, ambient energy, and productivity hypotheses. Orogenic processes and geological isolation may also influence current planthopper richness patterns.

## Figures and Tables

**Table 1:** The seven species richness hypotheses and their related environmental variables tested in this study.

Hypotheses	Environmental Variables
Ambient energy	Mean annual temperature (MAT)
	Annual potential evapotranspiration (PET)
Water–energy dynamics	Mean annual precipitation (MAP)
	Annual actual evapotranspiration (AET)
Productivity	Normalized difference vegetation index (NDVI)
	Net primary productivity (NPP)
Environmental stability	Temperature annual range (TAR)
	Precipitation seasonality (PS)
Habitat heterogeneity	Elevation range (ER)
	Topographical roughness (TR)
	Slope (SP)
Human activities	Human population (HPP)
	Gross domestic product (GDP)
Historical climate stability	Mean annual temperature change since the LGM (MAT anomaly)
	Mean annual precipitation change since the LGM (MAP anomaly)

**Table 2:** Univariate relationships between species richness and environmental variables using ordinary least squares (OLS) and spatial error simultaneous autoregressive (SAR_err_) models. Coefficients (coef), *R*^2^ (%), and Pseudo *R*^2^ (%) are shown. For the variable abbreviations, see Table 1.

	Species Richness			
	Coef_OLS_	*R* ^2^_OLS_	Coef_SAR_	Pseudo *R*^2^_SAR_
MAT	0.56	31.9 ***	0.45	52.6 ***
PET	−0.03	0.1 ns	−0.04	46 ns
MAP	0.57	33.4 ***	0.51	54 ***
AET	0.59	35.4 ***	0.55	54.9 ***
NDVI	0.46	21.3 ***	0.39	51.5 ***
NPP	0.55	30.3 ***	0.48	54.2 ***
TAR	−0.54	29.6 ***	−0.46	52.4 ***
PS	−0.37	13.8 ***	−0.28	48.8 ***
ER	0.12	1.6 ***	0.17	47.6 ***
TR	0.05	0.2 ns	0.15	46.1 ns
SP	0.18	3.5 ***	0.18	47.5 ***
HPP	0.44	19.4 ***	0.36	50.7 ***
GDP	0.38	14.6 ***	0.31	49.5 ***
MAT anomaly	−0.55	30.5 ***	−0.5	53.9 ***
MAP anomaly	0.06	0.4 ns	−0.01	45.9 ns

***p < 0.001; ns, not significant (*p* > 0.05).
